# Association between lipoprotein(a) and thromboembolism in patients with non-valvular atrial fibrillation: a cross-sectional study

**DOI:** 10.1186/s12944-022-01682-2

**Published:** 2022-08-25

**Authors:** Jie Song, Xiaoxue Zhang, Meng Wei, Yakun Bo, Xianhui Zhou, Baopeng Tang

**Affiliations:** 1 Department of Cardiac Pacing and Electrophysiology, The First Affiliated Hospital of Xinjiang Medical University, No. 137, Liyushan Road, Urumqi, 830054 PR China; 2 Xinjiang Key Laboratory of Cardiac Electrophysiology and Remodeling, The First Affiliated Hospital of Xinjiang Medical University, Urumqi, 830054 PR China; 3 Department of outpatient, The First Affiliated Hospital of Xinjiang Medical University, Urumqi, 830054 PR China

**Keywords:** Lipoprotein(a), Non-valvular atrial fibrillation, Ischemic stroke, Systemic embolism

## Abstract

**Background:**

Lipoprotein(a) [Lp(a)] is a recognized risk factor for ischemic stroke (IS); however, its role in thromboembolism in patients with non-valvular atrial fibrillation (NVAF) remains controversial. We aimed to assess the association of Lp(a) and IS and systemic embolism (SEE) in NVAF patients.

**Methods:**

In total, 16,357 patients with NVAF were recruited from the First Affiliated Hospital of Xinjiang Medical University from January 1, 2009, to December 31, 2021, and were divided into groups based on Lp(a) quartiles. Logistic regression models analyzed the association between Lp(a), IS, and SEE. The restriction cubic spline was used to assess the potential nonlinear relationship between Lp(a), IS, and SEE. We conducted subgroup analyses and estimated the multiplicative interaction between the stratified variables and Lp(a) to investigate whether the association between Lp(a) and IS and SEE was affected by age, sex, anticoagulants, and CHA_2_DS_2_-VASc score.

**Results:**

We identified 1319 IS and 133 SEE events. After correcting for CHA_2_DS_2_-VASc score and other potential confounders, each 1-standard deviation (SD) increase in log-Lp(a) was related to a 23% increased risk of IS (odds ratios [OR], 1.23; 95% confidence intervals [CI], 1.07–1.41). NVAF patients in the highest Lp(a) quartile were 1.23-fold more likely to have IS than those in the lowest quartile (OR, 1.23; 95% CI, 1.04–1.45). A positive linear relationship between Lp(a) and IS risk was observed (*P* for nonlinear = 0.341). In the fully adjusted model, subjects had a 1.78-fold increased risk of SEE for each 1-SD increase in log-Lp(a) (OR, 2.78; 95% CI, 1.78–4.36). Subjects in the highest Lp(a) quartile had a 2.38-fold elevated risk of SEE (OR, 3.38; 95% CI, 1.85–6.19) compared with the lowest quartile. Furthermore, Lp(a) had a nonlinear relationship with the risk of SEE (*P* for nonlinear = 0.005).

**Conclusions:**

Elevated Lp(a) concentration was significantly associated with IS and SEE, suggesting that Lp(a) may be an emerging biomarker that can help clinicians identify patients at high risk of thromboembolism in this population.

## Background

Atrial fibrillation (AF) is the most common supraventricular tachyarrhythmias, which increases the risk of ischemic stroke (IS) by 4–5-fold; and the morbidity and mortality of AF-related IS are both high [1]. Moreover, AF increases the risk of systemic embolism (SEE) with an annual incidence of 0.24% [2]. Owing to the prethrombotic state of AF, the management of this population focuses on the prevention of thromboembolism [3]. The CHA_2_DS_2_-VASc scoring system is a useful tool used in clinical practice to evaluate the risk of thromboembolism in AF patients [4]. However, the score still has limitations and does not fully consider potential risk factors such as blood lipids and obesity [5, 6]. Furthermore, there is increasing evidence that, in addition to the traditional atherogenic role of serum lipoprotein, it promotes thrombosis by affecting platelets and the coagulation system [7, 8]. Hence, lipoprotein can be expected to promote thromboembolism when considering its effect on the clotting function [3].

In fact, several studies have confirmed that low-density lipoprotein cholesterol (LDL-C) is closely relevant to IS in patients with AF [9, 10]. Lipoprotein(a) [Lp(a)] is a special form of LDL-C; it is composed of apolipoprotein B-100 containing LDL-like particle and apolipoprotein(a) through disulfide bonds [11–13], and is considered as an independent risk factor for IS [14, 15]. Previously, a prospective multicenter cohort study showed that Lp(a) was a risk factor for large artery atherosclerotic IS, and each one-unit increase in log_10_-Lp(a) was related to a 48% increase in stroke risk [14]. Nevertheless, it is not clear whether Lp(a) has the same effect in patients with AF. Okura et al. reported that Lp(a) levels were higher in patients with AF with cardiogenic stroke than in those without cardiogenic stroke [16]. A small case-control study reported that 48% of the patients with Lp(a) ≥ 30 mg/dL developed left atrial thrombosis. Subsequent multifactorial analysis confirmed that Lp(a) is a risk factor for thromboembolism in patients with chronic non-valvular atrial fibrillation (NVAF). This suggests that Lp(a) may be an emerging biomarker for identifying a high risk of thromboembolism in patients with AF [17, 18]. Aroniset et al. subsequently found that an Lp(a) > 50 mg/dL was related to an increased risk of IS; nevertheless, the same results were not obtained in patients with AF [19]. Although the above studies are representative to some extent, the following shortcomings should not be ignored: the small sample size, Asian populations were not included, and data from Chinese study populations were lacking. Most importantly, the existing literature suggests that the relationship between Lp(a) and AF thromboembolic events remains controversial. Therefore, the present research aimed to explore the association of Lp(a) and IS and SEE in patients with NVAF, and provide new ideas for the comprehensive clinical treatment and management of AF patients.

## Methods

### Study design and participants

This cross-sectional research was approved by the First Affiliated Hospital of Xinjiang Medical University Ethics Committee (ethics number: K202204–04). As this study was retrospective in nature, the ethics committee agreed to exempt patients from written informed consent.

**Fig. 1:**
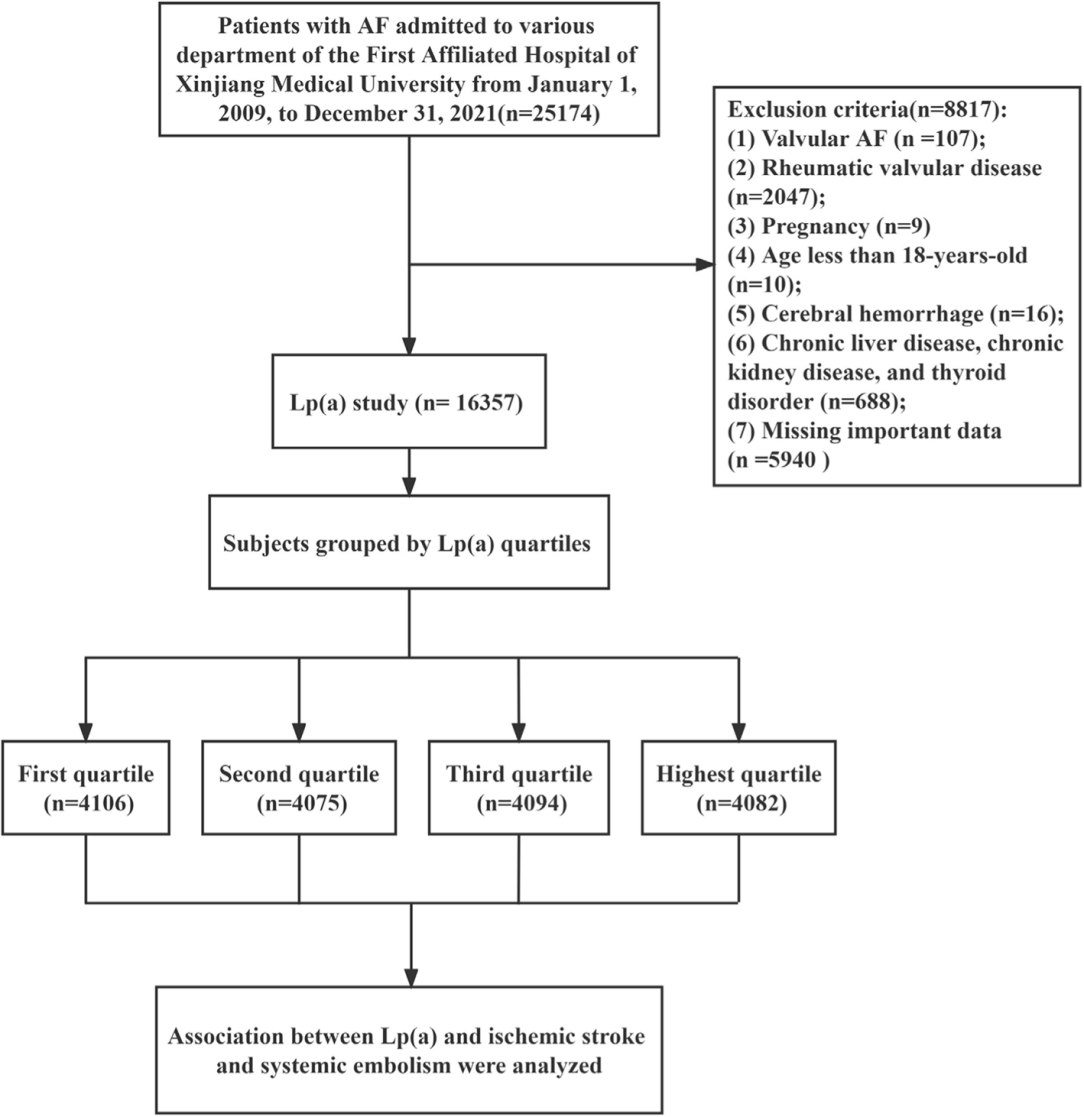
Flow chart for selecting the study population. AF, atrial fibrillation; Lp(a), Lipoprotein(a)

Patients with AF were identified by medical history, 12-lead electrocardiography, or 24-h Holter monitoring. Patients with AF admitted to all clinical departments of the First Affiliated Hospital of Xinjiang Medical University from January 1, 2009, to December 31, 2021, were continuously enrolled. Exclusion criteria of the study subjects were as follows: valvular AF (after mechanical valve replacement, and moderate and severe mitral stenosis complicated with AF), age < 18 years, rheumatic valvular disease, pregnant women, cerebral hemorrhage, chronic liver disease, chronic kidney disease, thyroid disorder, and missing Lp(a) and other important data. Chronic liver disease was defined as liver injury (≥ 6 months), including alcoholic liver disease, nonalcoholic fatty liver disease, metabolic fatty liver disease, and drug-induced liver injury [20]. Chronic kidney disease (CKD) was defined as kidney damage (presented as albuminuria, or determined by radiological or histological evidence) or decreased renal function (glomerular filtration rate < 60 ml/min/1.73 m^2^) for at least 3 months [21]. The reason for excluding CKD and chronic liver disease is that previous studies have shown that both these diseases can affect Lp(a) levels [22, 23], and CKD can crosstalk the association between Lp(a) and thromboembolism [24]. Finally, 16,357 patients with NVAF were enrolled in this study (Fig. 1).

### Data collection

We systematically reviewed the medical records of all subjects using the hospital system’s electronic medical record, including demographic data (age, sex, ethnicity), comorbidities (heart failure [HF], diabetes, hypertension, previous stroke, and history of vascular disease), medication history (oral anticoagulants, antiplatelet agents, and lipid-lowering agents), and CHA_2_DS_2_-VASc score (scoring criteria were as follows: hypertension, HF, 65–74 years, diabetes, vascular disease, and females each scored one point; age more than 75 years, and previous stroke/transient ischemic attack/thromboembolism scored two points, respectively).

Fasting venous blood was collected the next morning after hospitalization, and white, neutrophil, mononuclear, lymphocyte, and platelet counts; red blood cell distribution width (RDW); and Lp(a), apolipoprotein A1 (apoA-1), apolipoprotein B (apo B), total cholesterol [TC], triglycerides [TG], LDL-C, and high-density lipoprotein cholesterol [HDL-C]) were detected using an automated blood analyzer.

### Assessments of IS and SEE

Based on rigorous neurological physical examination and imaging (brain computed tomography [CT] or magnetic resonance imaging [MRI]), IS is defined as a focal neurological deficit [25]. A few patients had total facial nerve impairment, and those with lesions responsible for brain CT or MRI, or symptoms/signs lasting for more than 24-h, and events of cerebral hemorrhage were excluded by imaging examination [25]. SEE refers to sudden vascular insufficiency associated with arterial occlusion confirmed by clinical or radiological evidence (CT angiography or magnetic resonance angiography), including limb arterial, mesenteric artery, and renal artery embolisms [26, 27]. The diagnostic records of IS and SEE were confirmed according to the hospital’s electronic medical record system.

### Statistical analysis

According to Lp(a) quartiles, the recruited patients were divided into four groups (first quartile [Q1]: < 7.1 mg/dL, second quartile [Q2]: 7.1–13.4 mg/dL, third quartile [Q3]: 13.4–25.4 mg/dL, and highest quartile [Q4]: ≥ 25.4 mg/dL) to describe the baseline characteristics. The Kolmogorov Smirnov (KS) test was used to test for normality, and a *P* > 0.05 indicated conformity with normal distribution. Continuous variables are shown as mean ± standard deviation (SD) or median (interquartile range) based on data distribution type. One-way ANOVA or Kruskal-Wallis H test was utilized to compare between groups. The classified data are expressed as frequency (percentage), and the chi-square (*χ*^*2*^) test was used to compare the differences between groups. Lp(a) was used as the classification variable divided by quartiles and the continuous variable of logarithmic transformation (base 10) to fit the binary logistic regression model. Binary logistic regression analysis was performed to explore the association of Lp(a) and IS and SEE in patients with NVAF and to adjust for potential confounding factors to obtain the adjusted odds ratio (OR) and 95% confidence interval (CI). In the multivariate analysis, HF, hypertension, diabetes, previous stroke, and vascular disease history were not included as covariates alone because these indicators were variables in the CHA_2_DS_2_-VASc score. Model 1 was corrected for age, sex, and ethnicity. Model 2 was corrected for CHA_2_DS_2_-VASc score. Model 3 was corrected for ethnicity, CHA_2_DS_2_-VASc score, anticoagulant use, lipid-lowering drugs, LDL-C, HDL-C, apoA-1, apo B, TC, TG, and RDW. Subgroup analyses were utilized to further identify the relationship between Lp(a) and IS and SEE based on age (< 65 years, ≥ 65 years), sex, oral anticoagulants, and CHA_2_DS_2_-VASc score (< 2 or ≥ 2). The Wald test was used to estimate the multiplicative interaction between stratified variables and Lp(a). In addition, multivariable-corrected restriction cubic spline (RCS) was used to assess the potential nonlinear relationship between Lp(a), IS, and SEE. To consider the smoothness of the curve and avoid over-fitting in the principal spline curves of IS and SEE, according to Akaike’s information criterion (AIC), the number of knots corresponding to the minimum AIC value between 3 and 7 was selected [28]. Due to the retrospective and observational characteristics of this study, to avoid reverse causal correlation, we excluded patients who took statins 6 months before admission and subsequently conducted a sensitivity analysis to verify the robustness of the results.

All statistical analyses were performed using SPSS (version 21; SPSS Inc., Chicago, IL, USA) and R (version 4.1.1; R Foundation for Statistical Computing, Vienna, Austria). *P* <  0.05 (two-sided) was considered statistically different.

## Results

### Baseline characteristics

In this study, 16,357 patients with NVAF were enrolled, with an average age of 67.3 ± 12.4 years, of which 63.7% were male and 36.3% were female. The median Lp(a) concentration was 13.4 mg/dL (7.1–25.4 mg/dL). With the increase in Lp(a) level, the CHA_2_DS_2_-VASc score, white blood cell, neutrophil, monocyte, and platelet counts, and TC and LDL-C concentrations increased (Table 1). However, the percentage of subjects taking anticoagulants decreased with increasing Lp(a) levels. The percentage of patients receiving lipid-lowering drugs in the Lp(a) highest quartile group (Q4) was higher than those in the Q2 and Q3 groups.

**Table 1:** Baseline characteristics of patients with nonvalvular atrial fibrillation grouped by Lp(a) quartiles

Characteristics	Lp(a) quartiles, mg/dL				*χ*^*2*^/F/H	*P* value
	Q1(*n* = 4106)	Q2 (*n* = 4075)	Q3 (*n* = 4094)	Q4 (*n* = 4082)		
	< 7.1	[7.1, 13.4)	[13.4, 25.4)	≥ 25.4		
Age, year	68.0 (58.0, 76.0)	69.00 (59.0, 77.0)^a^	70.0 (60.0, 77.0)^b^	70.0 (60.0, 77.0)^c^	44.173	< 0.001
Women, n (%)	1462 (35.6)	1522 (37.4)	1455 (35.5)	1496 (36.7)	4.033	0.258
Ethnicity, n (%)						
Other	1078 (26.3)	1130 (27.7)	1112 (27.2)	1317 (32.3)		
Han	3028 (73.8)	2945 (72.3)	2982 (72.8)	2765 (67.7)^cef^	43.275	< 0.001
AF type, n (%)					53.770	< 0.001
Paroxysmal	1441 (35.1)	1326 (32.5)	1300 (31.8)^b^	1320 (32.3)^c^		
Persistent	282 (6.9)	183 (4.5)^a^	176 (4.3)^b^	222 (5.4)^c^		
Other	2383 (58.0)	2566 (63.0)^a^	2618 (63.9)^b^	2540 (62.2)^c^		
Heart failure, n (%)	281 (6.8)	319 (7.8)	305 (7.5)	323 (7.9)	4.195	0.241
Hypertension, n (%)	2259 (55.0)	2320 (56.9)	2301 (56.2)	2301 (56.4)	3.235	0.357
Diabetes mellitus, n (%)	842 (20.5)	768 (18.9)	838 (20.5)	811 (19.9)	4.608	0.203
Stroke, n (%)	107 (2.6)	112 (2.8)	119 (2.9)	126 (3.1)	1.907	0.592
Vascular disease, n (%)	191 (4.7)	180 (4.4)	158 (3.9)	211 (5.2)^f^	8.382	0.039
CHA_2_DS_2_-VASc score	2.2 ± 1.4	2.3 ± 1.4^a^	2.3 ± 1.4^b^	2.3 ± 1.4^c^	7.410	< 0.001
WBC count, 10^9^/L	6.7 (5.3, 8.2)	6.8 (5.6, 8.5)^a^	6.9 (5.6, 8.4)^b^	7.1 (5.7, 8.7)^cef^	74.448	< 0.001
Neutrophil count,10^9^/L	4.0 (3.0, 5.8)	4.3 (3.2, 6.4)^a^	4.4 (3.3, 6.5)^b^	4.6 (3.4, 6.9)^cef^	165.592	< 0.001
Monocyte count, 10^9^/L	0.5 (0.4,0.7)	0.6 (0.4, 0.8)^a^	0.6 (0.4, 0.8)	0.6 (0.4, 0.8)^cef^	69.262	< 0.001
Lymphocyte count, 10^9^/L	1.8 (1.4, 2.3)	1.8 (1.4, 2.3)	1.8 (1.4, 2.3)	1.8 (1.4, 2.3)	8.581	0.035
Platelet count, 10^9^/L	191.0 (148.0, 239.0)	200.0 (150.0, 247.0)^a^	201.0 (157.0, 248.0)^b^	210.0 (167.0, 262.0)^cef^	156.017	< 0.001
RDW, %	13.5 (12.9, 14.6)	13.7 (13.0, 14.8)^a^	13.7 (13.0, 14.8)^b^	13.7 (13.0, 14.8)^c^	50.128	< 0.001
TC, mmol/L	3.4 (2.8, 4.1)	3.5 (2.9, 4.3)^a^	3.6 (3.0, 4.3)^bd^	3.8 (3.1, 4.5)^cef^	225.102	< 0.001
TG, mmol/L	1.2 (0.9, 1.8)	1.2 (0.9, 1.7)	1.2 (0.9, 1.6)^b^	1.3 (0.9, 1.8)^cef^	60.614	< 0.001
LDL-C, mmol/L	2.1 (1.6, 2.7)	2.2 (1.7, 2.8)^a^	2.3 (1.8, 2.9)^bd^	2.4 (1.9, 3.0)^cef^	282.551	< 0.001
HDL-C, mmol/L	1.0 (0.8, 1.2)	1.0 (0.8, 1.3)	1.1 (0.9, 1.3)^bd^	1.0 (0.9, 1.3)^c^	28.420	< 0.001
apoA-1, g/L	1.1 (1.0, 1.3)	1.1 (1.0, 1.3)	1.1 (1.0, 1.3)	1.1 (1.0, 1.3)	5.934	0.115
apo B, g/L	0.7 (0.6, 0.9)	0.7 (0.6, 0.9)^a^	0.8 (0.6, 0.9)^bd^	0.8 (0.7, 1.0)^cef^	319.269	< 0.001
Lipid-lowering medications, n (%)	1063 (25.9)	926 (22.7)^a^	899 (22.0)	1051 (25.8)^ef^	27.728	< 0.001
Antiplatelet agents, n (%)	811 (19.8)	776 (19.0)	809 (19.8)	818 (20.0)	1.401	0.705
Anticoagulants, n (%)	1194 (29.1)	962 (23.6)^a^	861 (21.0)^bd^	890 (21.8)^c^	89.187	< 0.001
NOAC	684 (57.3)	483 (50.2)^a^	419 (48.7)^b^	510 (57.3)	24.316	< 0.001
Warfarin	510 (42.7)	479 (49.8)^a^	442 (51.3)^b^	380 (42.7)		

*Abbreviations*: *AF* Atrial fibrillation*, Lp(a)* Lipoprotein(a), *HDL-C* High-density lipoprotein cholesterol, *LDL-C* Low-density lipoprotein cholesterol, *TC* Total cholesterol, *TG* Triglycerides, *RDW* Red-cell distribution width, *apoA-I* Apolipoprotein A1, *apo B* Apolipoprotein B, *NOAC* New oral anticoagulants

^a^indicates a significant difference between Q1 and Q2

^b^indicates a significant difference between Q1 and Q3

^c^indicates a significant difference between Q1 and Q4

^d^indicates a significant difference between Q2 and Q3

^e^indicates a significant difference between Q2 and Q4

^f^indicates a significant difference between Q3 and Q4

### Association of Lp(a) and IS

**Fig. 2:**
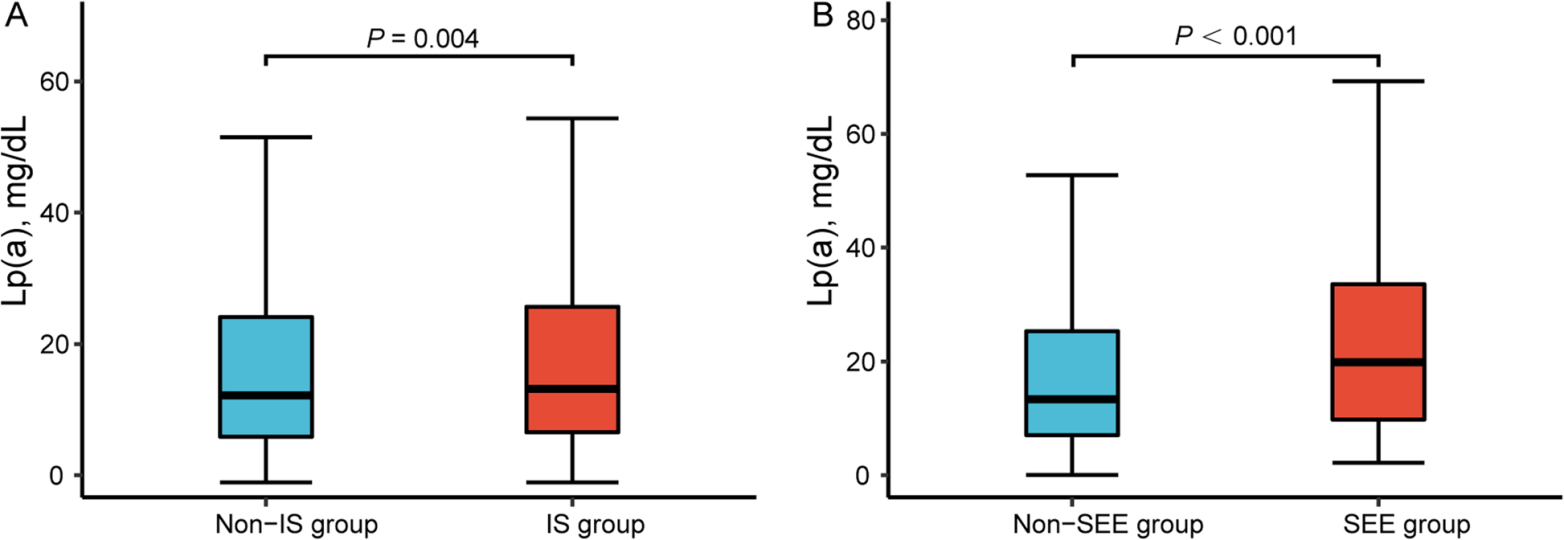
Comparison of lipoprotein (a) [Lp(a)] levels between groups. **A** The level of Lp(a) in ischemic stroke (IS) group was significantly higher than non-IS group (*P* = 0.004); **B** The level of Lp(a) in systemic embolism (SEE) group was significantly higher than non-IS group (*P* < 0.001)

**Fig. 3:**
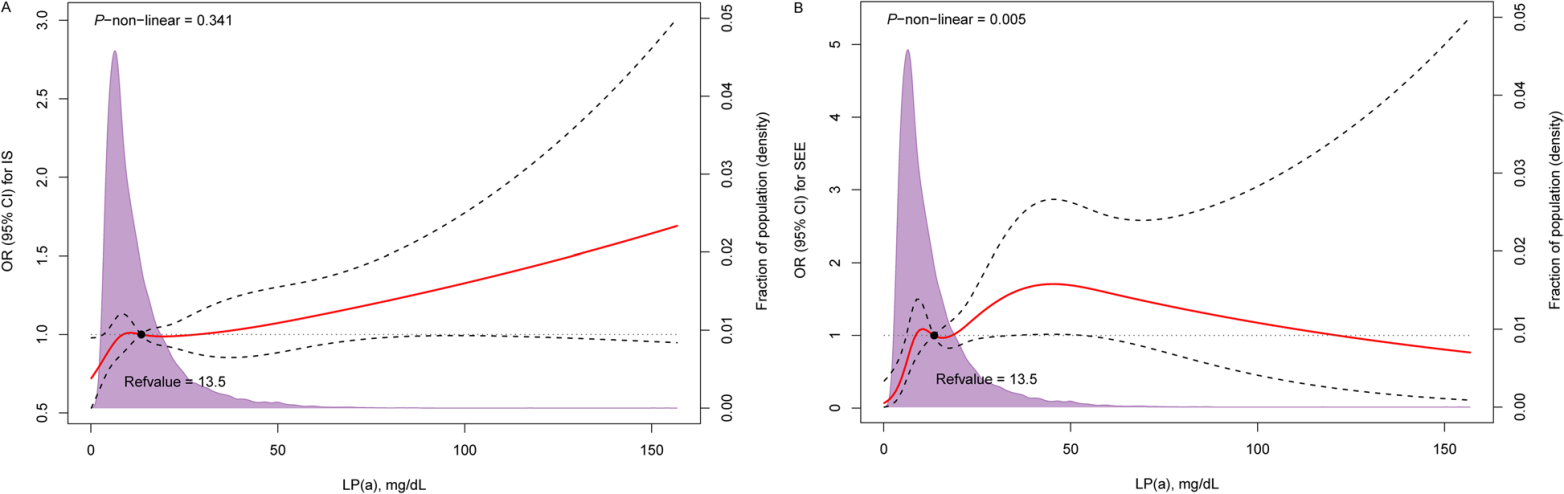
Multivariable-adjusted restricted cubic spline analysis for the dose-response relationship between lipoprotein (a) [Lp(a)] and risk of ischemic stroke (IS) and systemic embolism (SEE). The solid red line represents the odds ratio (OR) and the dashed lines represent the 95% confidence intervals (95% CI). Knots at the 10th, 50th, and 90th percentiles. The purple curve represents the per cent of the density distribution of Lp(a) in the study population (right y-axis). The model was adjusted for ethnicity, CHA_2_DS_2_-VASc score, anticoagulants, lipid-lowering medications, triglycerides, total cholesterol, high-density lipoprotein cholesterol, low-density lipoprotein cholesterol, apolipoprotein A1, apolipoprotein B, and red-cell distribution width

IS occurred in 1319 patients with NVAF, with a total prevalence rate of 8.1%. As shown in Fig. 2A, the Lp(a) level was significantly higher in patients with IS than in patients without IS. The results of the univariable and multivariable logistic analyses of IS in patients with different Lp(a) levels and NVAF are shown in Table 2. With an increase in Lp(a) levels, the prevalence of IS increased (trend *χ*^*2*^, *P* = 0.007). Because Lp(a) presents a highly biased distribution, was transformed into logarithmic rank [log-Lp(a)] and fitted to the regression model as a continuous variable. Univariate logistic analysis showed that for each one SD increase in log-Lp(a), the risk of IS in patients with NVAF increased by 25% (OR, 1.25; 95% CI, 1.09–1.43). After correcting for ethnicity, CHA_2_DS_2_-VASc score, anticoagulants, lipid-lowering drugs, LDL-C, HDL-C, apoA-1, apo B, TC, TG, and RDW, log-Lp(a) increased the risk of IS by 23% for each one increased SD (OR, 1.23; 95% CI, 1.07–1.41). After converting Lp(a) into classification variables according to the quartiles, NVAF patients with Lp(a) highest quartile (Q4 ≥ 25.4 mg/dL) had a 23% increased risk of IS (OR, 1.23; 95% CI, 1.04–1.45) compared with those with Lp(a) <  7.1 mg/dL in the fully corrected model. The dose-response curve of Lp(a) and IS is shown in Fig. 3A. A positive linear relationship between Lp(a) and IS risk was found (*P* for nonlinear = 0.341).

**Table 2:** Ischemic stroke risk of different lipoprotein (a) levels (mg/dL) in patients with non-valvular atrial fibrillation

Lp (a)	Events, n (%)	Crude model		Model1		Model 2		Model 3	
		OR (95% CI)	*P*	OR (95% CI)	*P*	OR (95% CI)	*P*	OR (95% CI)	*P*
Q1 (< 7.1)	289 (7.0)	1.00		1.00		1.00		1.00	
Q2 [7.1, 13.4)	335 (8.2)	1.18 (1.00, 1.39)	0.044	1.14 (0.96, 1.34)	0.126	1.15 (0.97, 1.36)	0.110	1.13 (0.95, 1.34)	0.161
Q3 [13.4, 25.4)	338 (8.3)	1.19 (1.01, 1.40)	0.038	1.14 (0.97, 1.34)	0.125	1.16 (0.98, 1.37)	0.084	1.14 (0.96, 1.35)	0.124
Q4 (≥ 25.4)	357 (8.8)	1.27 (1.08, 1.49)	0.004	1.21 (1.03, 1.42)	0.024	1.22 (1.03, 1.43)	0.021	1.23 (1.04, 1.45)	0.013
*P* for trend	0.007^a^		0.034		0.150		0.128		0.122
Log-Lp(a)		1.25 (1.09, 1.43)	0.001	1.20 (1.05, 1.37)	0.009	1.21 (1.05, 1.39)	0.007	1.23 (1.07, 1.41)	0.004

*Abbreviations*: *Lp(a)* Lipoprotein(a), *Log* log-transformed, *OR* Odds ratio, *95% CI* 95% confidence interval

Model 1: adjusted for sex, age, and ethnicity; Model 2: adjusted for CHA_2_DS_2_-VASc score; Model 3: adjusted for ethnicity, CHA_2_DS_2_-VASc score, anticoagulants, lipid-lowering medications, triglycerides, total cholesterol, high-density lipoprotein cholesterol, low-density lipoprotein cholesterol, apolipoprotein A1, apolipoprotein B, and red-cell distribution width

^a^Mantel-Haenszel *χ*^*2*^ test

### Association of Lp(a) and SEE

SEE occurred in 133 patients with NVAF, with an overall prevalence of 0.8%. The Lp(a) concentration was significantly higher in patients with SEE than that in subjects without SEE (Fig. 2B). The results of the univariable and multivariable logistic analyses of SEE in patients with different Lp(a) levels and NVAF are shown in Table 3. Compared with Q1, the prevalence of SEE was higher in the Q2, Q3, and Q4 Lp(a) groups (all *P* <  0. 05). Univariable analysis revealed that for each additional SD of log-Lp(a), the risk of SEE in patients with NVAF increased 1.66-fold (OR, 2.66; 95% CI, 1.73–4.08). In the fully corrected model, the risk of SEE increased 1.78-fold for each additional SD of log-Lp(a) (OR, 2.78; 95% CI, 1.78–4.36). After dividing Lp(a) into classification variables according to quartiles, in the fully corrected model, NVAF patients with Lp(a) highest quartile (Q4 ≥ 25.4 mg/dL) had a 2.38-fold increased risk of SEE compared to patients with Lp(a) <  7.1 mg/dL (OR, 3.38; 95% CI, 1.85–6.19). In addition, the multivariable-corrected RCS model showed a nonlinear relationship between Lp(a) and SEE risk (*P* for nonlinear = 0.005) (Fig. 3B).

**Table 3:** Systemic embolism risk of different lipoprotein (a) levels (mg/dL) in patients with non-valvular atrial fibrillation

Lp(a)	Events, n (%)	Crude model		Model 1		Model 2		Model 3	
		OR (95% CI)	*P*	OR (95% CI)	*P*	OR (95% CI)	*P*	OR (95% CI)	*P*
Q1 (< 7.1)	14 (0.3)	1.00		1.00		1.00		1.00	
Q2 [7.1, 13.4)	37 (0.9)^a^	2.68 (1.45, 4.96)	0.002	2.59 (1.40, 4.80)	0.003	2.60 (1.40,4.82)	0.002	2.48 (1.33, 4.61)	0.004
Q3 [13.4, 25.4)	34 (0.8)^b^	2.45 (1.31, 4.57)	0.005	2.36 (1.26, 4.40)	0.007	2.39 (1.28, 4.47)	0.006	2.38 (1.27, 4.46)	0.007
Q4 (≥ 25.4)	48 (1.2)^c^	3.48 (1.92, 6.32)	< 0.001	3.27 (1.80, 5.95)	< 0.001	3.35 (1.84, 6.09)	< 0.001	3.38 (1.85, 6.19)	< 0.001
*P* for trend	< 0.001		0.001		0.002		0.001		0.001
Log-Lp(a)		2.66 (1.73, 4.08)	< 0.001	2.54 (1.65, 3.91)	< 0.001	2.59 (1.68, 4.00)	< 0.001	2.78 (1.78, 4.36)	< 0.001

*Abbreviations*: *Lp(a)* Lipoprotein(a), *Log* log-transformed, *OR* Odds ratio, *95% CI* 95% confidence interval

Model 1: adjusted for sex, age, and ethnicity; Model 2: adjusted for CHA_2_DS_2_-VASc score; Model 3: adjusted for ethnicity, CHA_2_DS_2_-VASc score, anticoagulants, lipid-lowering medications, triglycerides, total cholesterol, high-density lipoprotein cholesterol, low-density lipoprotein cholesterol, apolipoprotein A1, apolipoprotein B, and red-cell distribution width

^a^indicates a significant difference between Q1 and Q2

^b^indicates a significant difference between Q1 and Q3

^c^indicates a significant difference between Q1 and Q4

### Subgroup analysis

**Fig. 4:**
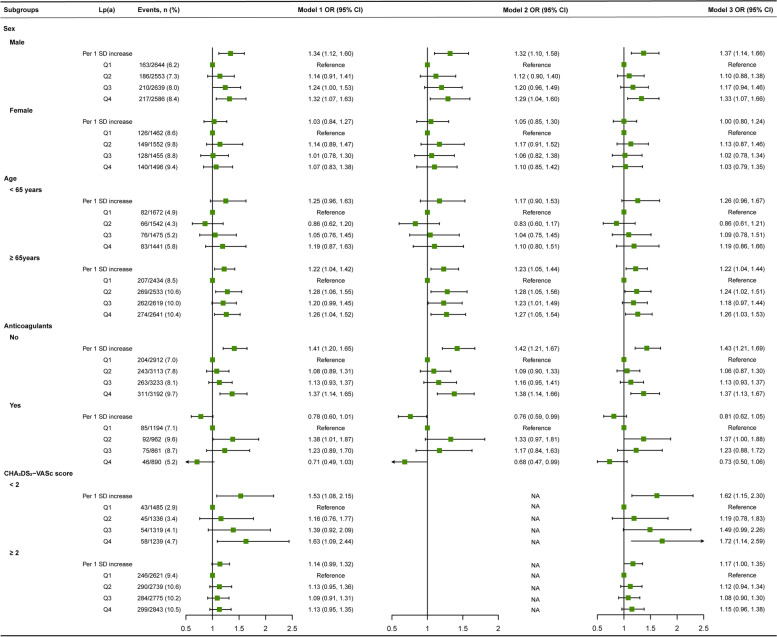
Subgroup analysis of Lp(a) on ischemic stroke in non-valvular atrial fibrillation patients. Note: Interactions of Lp(a) with sex, age, anticoagulants, and CHA_2_DS_2_-VASc score were *P* = 0.590, *P* = 0.216, *P* < 0.001, and *P* = 0.207 for Lp(a) categories and *P* = 0.113, *P* = 0.888, *P* < 0.001, and *P* = 0.078 for continuous Lp(a) [Log-Lp(a)], respectively. Model 1: adjusted for sex, age, and ethnicity; Model 2: adjusted for CHA_2_DS_2_-VASc score; Model 3: adjusted for ethnicity, CHA_2_DS_2_-VASc score, anticoagulants, lipid-lowering medications, triglycerides, total cholesterol, high-density lipoprotein cholesterol, low-density lipoprotein cholesterol, apolipoprotein A1, apolipoprotein B, and red-cell distribution width. Lp(a), Lipoprotein(a); Log, log-transformed; OR, odds ratio; CI, confidence interval

The results of the subgroup effects of IS are shown in Fig. 4. There was a significant multiplicative interaction between anticoagulants and Lp(a) (as a categorical variable and as a continuous variable of logarithmic transformation, respectively) in IS (*P* for interaction < 0.001). The IS adjusted OR value of NVAF patients without oral anticoagulants was 1.43 (95% CI, 1.21–1.69), but after adjusting for confounders in subjects with oral anticoagulants, the association between Lp(a) and IS was no longer statistically significant (OR, 0.81; 95% CI, 0.62–1.05). Moreover, patients with a CHA_2_DS_2_-VASc score of < 2 had a higher risk of stroke in the high Lp(a) group (OR, 1.72; 95% CI, 1.14–2.59).

**Fig. 5:**
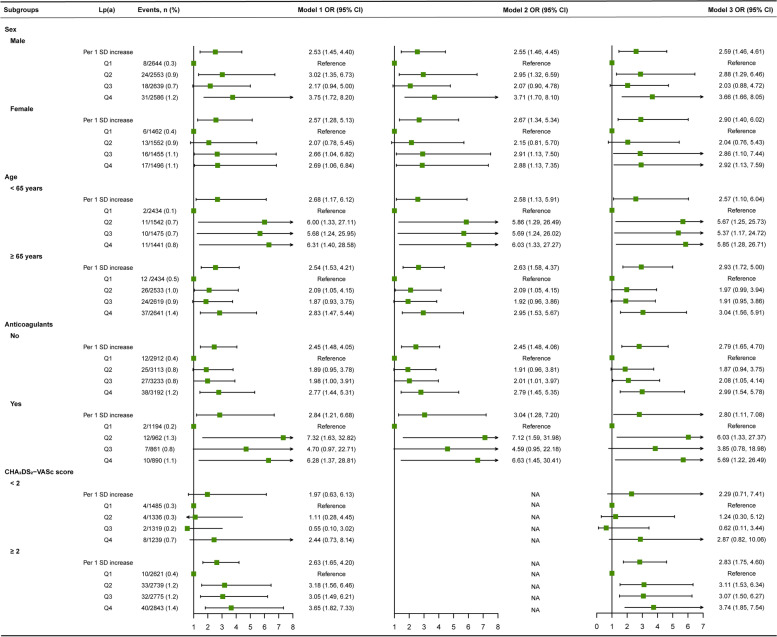
Subgroup analysis of Lp(a) on systemic embolism in non-valvular atrial fibrillation patients. Note: Interactions of Lp(a) with sex, age, anticoagulants, and CHA_2_DS_2_-VASc score were *P* = 0.461, *P* = 0.566, *P* = 0.408, and *P* = 0.261 for Lp(a) categories and *P* = 0.726, *P* = 0.967, *P =* 0.714, and *P* = 0.619 for continuous Lp(a) [Log-Lp(a)], respectively. Model 1: adjusted for sex, age, and ethnicity; Model 2: adjusted for CHA_2_DS_2_-VASc score; Model 3: adjusted for ethnicity, CHA_2_DS_2_-VASc score, anticoagulants, lipid-lowering medications, triglycerides, total cholesterol, high-density lipoprotein cholesterol, low-density lipoprotein cholesterol, apolipoprotein A1, apolipoprotein B, and red-cell distribution width. Lp(a), Lipoprotein(a); Log, log-transformed; OR, odds ratio; CI, confidence interval

The results of the subgroup analysis of SEE are shown in Fig. 5. No multiplicative interaction was observed between Lp(a) [as a continuous variable of logarithmic transformation and as a categorical variable] and sex, age, anticoagulants, CHA_2_DS_2_-VASc score. NVAF patients with a CHA_2_DS_2_-VASc score ≥ 2 had a higher risk of SEE in the highest quartile of Lp(a) (Q4 ≥ 25.4 mg/dL) (OR, 3.74; 95% CI, 1.85–7.54).

### Sensitivity analysis

Studies have reported that statins may affect Lp(a) levels, resulting in an increase in the levels [29]. Thus, we re-analyzed the association between Lp(a), IS, and SEE after excluding patients who received lipid-lowering drugs 6 months before admission. The outcomes of the sensitivity analysis are illustrated in Table 4 and Table 5, respectively. Even after adjusting for multiple covariates, high Lp(a) concentrations were associated with an increased risk of IS and SEE.

**Table 4:** Odds ratios (95% confidence intervals) for incident ischemic stroke of sensitivity analysis (excluded 3939 participants with lipid-lowering therapy)

Lp(a)	Crude OR (95%C I)	OR (95% CI)	OR (95% CI)	OR (95% CI)
		Model 1	Model 2	Model 3
Q1	Reference	Reference	Reference	Reference
Q2	1.22 (1.00, 1.49)	1.16 (0.95, 1.42)	1.16 (0.94,1.42)	1.15 (0.93, 1.41)
Q3	1.40 (1.16, 1.70)	1.32 (1.09, 1.61)	1.34 (1.10, 1.64)	1.33 (1.09, 1.63)
Q4	2.04 (1.70, 2.45)	1.93 (1.60, 2.32)	1.93 (1.59, 2.33)	1.95 (1.61, 2.37)
*P* for trend	< 0.001	< 0.001	< 0.001	< 0.001
Log-Lp(a)	1.97 (1.68, 2.31)	1.88 (1.60, 2.21)	1.88 (1.60, 2.22)	1.92 (1.63, 2.27)

*Abbreviations*: *Lp(a)* Lipoprotein(a), *Log* Log-transformed, *OR* Odds ratio, *95% CI* 95% confidence interval

Model 1: adjusted for sex, age, and ethnicity; Model 2: adjusted for CHA_2_DS_2_-VASc score; Model 3: adjusted for ethnicity, CHA_2_DS_2_-VASc score, anticoagulants, triglycerides, total cholesterol, high-density lipoprotein cholesterol, low-density lipoprotein cholesterol, apolipoprotein A1, apolipoprotein B, and red-cell distribution width

**Table 5:** Odds ratios (95% confidence intervals) for incident systemic embolism of sensitivity analysis (excluded 3939 participants with lipid-lowering therapy)

Lp(a)	Crude OR (95% CI)	OR (95% CI)	OR (95% CI)	OR (95% CI)
		Model 1	Model 2	Model 3
Q1	Reference	Reference	Reference	Reference
Q2	2.02 (1.04, 3.91)	1.94 (1.00, 3.76)	1.92 (0.99, 3.73)	1.84 (0.94, 3.58)
Q3	1.84 (0.94, 3.60)	1.75 (0.89, 3.44)	1.75 (0.89, 3.43)	1.80 (0.92, 3.55)
Q4	3.04 (1.62, 5.70)	2.85 (1.51, 5.35)	2.82 (1.50, 5.29)	3.01 (1.59, 5.72)
*P* for trend	0.005	0.01	0.011	0.006
Log-Lp(a)	2.93 (1.78, 4.82)	2.79 (1.69, 4.61)	2.76 (1.67, 4.56)	3.21 (1.90, 5.43)

*Abbreviations*: *Lp(a)* Lipoprotein(a), *Log* Log-transformed, *OR* Odds ratio, *95% CI* 95% confidence interval

Model 1: adjusted for sex, age, and ethnicity; Model 2: adjusted for CHA_2_DS_2_-VASc score; Model 3: adjusted for ethnicity, CHA_2_DS_2_-VASc score, anticoagulants, triglycerides, total cholesterol, high-density lipoprotein cholesterol, low-density lipoprotein cholesterol, apolipoprotein A1, apolipoprotein B, and red-cell distribution width

## Discussion

To our knowledge, this is the first cross-sectional research to assess the association of Lp(a) and the prevalence of thromboembolism in patients with NVAF. This research demonstrated that increased Lp(a) levels were associated with an elevated risk of IS and SEE in patients with NVAF, even after adjusting for confounding factors, such as age, sex, and CHA_2_DS_2_-VASc score. Notably, the RCS curve showed a positive linear relationship between Lp(a) and the risk of IS, and a nonlinear relationship between Lp(a) and the risk of SEE.

Few studies have been performed on the association of Lp(a) concentrations and AF-associated thromboembolic risk. Two previous small-sample clinical studies have shown that an increase in serum Lp(a) levels is closely related to thromboembolism [18] and left atrial thrombosis [17] in patients with AF, which is consistent with our results. Interestingly, however, a community-based cohort study indicated that elevated Lp(a) concentrations were associated with IS in the general population, but not in AF patients [19]; the reason for the inconsistency with our findings may be different sample sources. Due to the nature of observational research, more multicenter and prospective studies are warranted in the future to verify the current research results.

In the present study, we found that oral anticoagulants was a significant effect modifier of IS, indicating that the independent effect of Lp(a) on IS is affected by anticoagulants. Lp(a) was less associated with stroke in patients with NVAF receiving anticoagulants therapy, whereas it had a stronger correlation with IS in NVAF patients without oral anticoagulants. Hence, combined with the results of this study, we can speculate that the concentration of Lp(a) may play a critical role in the occurrence and development of IS; nevertheless, more clinical and experimental studies are required to prove the effect of Lp(a) on IS. In future clinical studies, we should follow the recommendations of AF guidelines and recommend that patients with NVAF at high risk of stroke receive oral anticoagulants. Meanwhile, considering that Asian AF patients are more prone to IS than non-Asians even if they receive anticoagulation therapy [1], and even though treatment with oral anticoagulants reduces the stroke risk by 60% in patients with AF, patients with AF still have a high residual cardiovascular risk [30]. We should therefore pay attention to the concentration of serum lipoprotein in patients with AF receiving anticoagulant therapy, and in addition to statins, we can consider increasing non-statins to reduce lipids if necessary. Conventional LDL-C and apo B reduction therapy had little effect on Lp(a) levels, whereas the proprotein-converting enzyme subtilisin/Kexin 9 (PCSK-9) monoclonal antibodies not only reduced 50–60% LDL-C on average, but also reduced Lp(a) by 25–30% [31, 32]. Thus far, targeted treatment to reduce Lp(a) is still under development, and more prospective clinical studies are needed to verify that reducing the level of Lp(a) can reduce thromboembolism risk in patients with AF. According to the ESC AF guidelines, CHA_2_DS_2_-VASc < 2 scores are considered to indicate a low risk of stroke, and these patients should decide whether to use anticoagulant therapy to prevent thromboembolism based on individual characteristics and patient wishes [4]. In this study, we observed that an increased level of Lp(a) was associated with a higher risk of IS in AF patients with CHA_2_DS_2_-VASc < 2 scores, indicating that the residual cardiovascular risk in this population remains high, and clinicians should focus on it. A small-sample case-control study showed that Lp(a) was an independent risk factor for thromboembolism in NVAF patients with a CHA_2_DS_2_-VASc score of 0–1 [33]. However, they did not report the respective prevalence rates of IS and SEE in thromboembolic events and the results of logistic regression analysis; therefore, it cannot be concluded that Lp(a) is related to IS and SEE in patients with NVAF with low CHA_2_DS_2_-VASc scores. Interestingly, the results of this study indicated a stronger association between Lp(a) and SEE in subjects with CHA_2_DS_2_-VASc ≥2 scores, even if CHA_2_DS_2_-VASc was not observed as the effect modifier of this association. SEE is more likely to occur in senile patients with AF [34]. Besides, in our research, the prevalence of SEE was significantly higher in patients with CHA_2_DS_2_-VASc ≥2 scores than in those with CHA_2_DS_2_-VASc < 2 scores (1.1% VS. 0.3%), and the average age of subjects with CHA_2_DS_2_-VASc ≥2 scores was higher than that of subjects with CHA_2_DS_2_-VASc < 2 (72.8 ± 9.3 years VS. 56.1 ± 10.0 years). Consequently, the relationship between the level of Lp(a) and SEE in NVAF patients with CHA_2_DS_2_-VASc ≥2 scores goes beyond traditional risk factors. In patients with a high risk of thromboembolism, the concentration of Lp(a) may be a therapeutic target to which clinicians should pay attention.

The pathophysiological mechanism by which Lp(a) increases the risk of thromboembolism in NVAF patients is still less clear cut, and the possible mechanisms are as follows. First, the structure of apolipoprotein B-100 in Lp(a) is similar to that of LDL-C [19, 35], which can enhance endothelial cell adhesion and molecular expression, interfere with vascular permeability, and promote foam cell formation, thus causing arteriosclerosis [36, 37]. Second, Lp(a), similar to fibrinogen, can weaken platelet-mediated fibrinolysis by interfering with the binding of fibrinogen to the platelet surface, thus promoting thrombosis [11, 38–40]. Finally, Lp(a) is the main carrier of oxidized phospholipid, which has important pro-inflammatory and atherosclerotic effects, and triggers the inflammatory reaction of arterial wall by promoting macrophage apoptosis and the secretion of inflammatory factors [41–44]. In short, atherosclerosis, vessel wall inflammation, and thrombosis may lead to thromboembolism, but the specific mechanism remains unclear. In the future, more in vivo experiments involving an increase in Lp(a) may be needed to strengthen the discussion of this mechanism.

This study has several limitations. First, owing to the limitations of data collection, the present study could not be stratified according to the classification of IS, so this study could not infer that Lp(a) was related to cardiogenic stroke in NVAF patients. In the future, we plan to carry out a multi-center, prospective cohort study to further explore whether Lp(a) is an intervention target for preventing cardiogenic stroke in NVAF patients. Second, this was a cross-sectional study, which can only provide etiological clues and cannot make causal inferences. Third, although multiple potential covariates were corrected, the possibility of residual confounding cannot be ruled out. Finally, our data come from the Chinese population in Asia, so the conclusion cannot be extended to other countries and ethnic groups.

## Conclusions

Elevated Lp(a) concentration was significantly related to IS and SEE. Lp(a) may be an emerging biomarker to help clinicians identify the high risk of thromboembolism in this population. These findings provide guidance for strategies aimed at reducing Lp(a) levels to prevent adverse cardiovascular events in patients with AF.

## Data Availability

The datasets used and/or analysed during the current study are available from the corresponding author on reasonable request.

## Abbreviations

AF: Atrial fibrillation
apoA-1: apolipoprotein A1
apo B: apolipoprotein B
CT: computed tomography
HF: heart failure
HDL-C: high-density lipoprotein cholesterol
IS: ischemic stroke
LDL-C: low-density lipoprotein cholesterol
Lp(a): lipoprotein(a)
MRI: magnetic resonance imaging
NVAF: non-valvular atrial fibrillation
RDW: red blood cell distribution width
SEE: Systemic embolism
SD: Standard deviation
TC: Total cholesterol

## References

[1] Chiang CE, Okumura K, Zhang S, Chao TF, Siu CW, Wei Lim T (2017). 2017 consensus of the Asia Pacific Heart Rhythm Society on stroke prevention in atrial fibrillation. J Arrhythm.

[2] Bekwelem W, Connolly SJ, Halperin JL, Adabag S, Duval S, Chrolavicius S (2015). Extracranial systemic embolic events in patients with nonvalvular atrial fibrillation: incidence, risk factors, and outcomes. Circulation.

[3] Ding WY, Protty MB, Davies IG, Lip GYH (2022). Relationship between lipoproteins, thrombosis, and atrial fibrillation. Cardiovasc Res.

[4] Kirchhof P, Benussi S, Kotecha D, Ahlsson A, Atar D, Casadei B (2016). 2016 ESC guidelines for the management of atrial fibrillation developed in collaboration with EACTS. Eur Heart J.

[5] Sulzgruber P, Wassmann S, Semb AG, Doehner W, Widimsky P, Gremmel T (2019). Oral anticoagulation in patients with non-valvular atrial fibrillation and a CHA_2_DS_2_-VASc score of 1: a current opinion of the European Society of Cardiology Working Group on cardiovascular pharmacotherapy and European Society of Cardiology Council on Stroke. Eur Heart J Cardiovasc Pharmacother.

[6] Jagadish PS, Kabra R (2019). Stroke risk in atrial fibrillation: beyond the CHA_2_DS_2_-VASc score. Curr Cardiol Rep.

[7] Ouweneel AB, Van Eck M (2016). Lipoproteins as modulators of atherothrombosis: from endothelial function to primary and secondary coagulation. Vasc Pharmacol.

[8] Deguchi H, Elias DJ, Griffin JH (2017). Minor plasma lipids modulate clotting factor activities and may affect thrombosis risk. Res Pract Thromb Haemost.

[9] Qi Z, Chen H, Wen Z, Yuan F, Ni H, Gao W (2017). Relation of low-density lipoprotein cholesterol to ischemic stroke in patients with nonvalvular atrial fibrillation. Am J Cardiol.

[10] Liu W, Xiong N, Xie K, Wu B, Qi Z, Zhou P (2020). A stricter control of low-density lipoprotein is necessary for thrombosis reduction in “lower thrombosis risk” patients with atrial fibrillation: a multicenter retrospective cohort study. J Thromb Thrombolysis.

[11] Cegla J, Neely RDG, France M, Ferns G, Byrne CD, Halcox J (2019). HEART UK consensus statement on lipoprotein(a): a call to action. Atherosclerosis.

[12] Gencer B, Kronenberg F, Stroes ES, Mach F (2017). Lipoprotein(a): the revenant. Eur Heart J.

[13] Schmidt K, Noureen A, Kronenberg F, Utermann G (2016). Structure, function, and genetics of lipoprotein (a). J Lipid Res.

[14] Arnold M, Schweizer J, Nakas CT, Schutz V, Westphal LP, Inauen C (2021). Lipoprotein(a) is associated with large artery atherosclerosis stroke aetiology and stroke recurrence among patients below the age of 60 years: results from the BIOSIGNAL study. Eur Heart J.

[15] Emerging Risk Factors C, Erqou S, Kaptoge S, Perry PL, Di Angelantonio E, Thompson A (2009). Lipoprotein(a) concentration and the risk of coronary heart disease, stroke, and nonvascular mortality. JAMA.

[16] Okura H, Inoue H, Tomon M, Nishiyama S, Yoshikawa T (1998). Increased plasma lipoprotein(a) level in cardioembolic stroke with non-valvular atrial fibrillation. Intern Med.

[17] Igarashi Y, Yamaura M, Ito M, Inuzuka H, Ojima K, Aizawa Y (1998). Elevated serum lipoprotein(a) is a risk factor for left atrial thrombus in patients with chronic atrial fibrillation: a transesophageal echocardiographic study. Am Heart J.

[18] Igarashi Y, Kasai H, Yamashita F, Sato T, Inuzuka H, Ojima K (2000). Lipoprotein(a), left atrial appendage function and thromboembolic risk in patients with chronic nonvalvular atrial fibrillation. Jpn Circ J.

[19] Aronis KN, Zhao D, Hoogeveen RC, Alonso A, Ballantyne CM, Guallar E (2017). Associations of lipoprotein(a) levels with incident atrial fibrillation and ischemic stroke: the ARIC (atherosclerosis risk in communities) study. J Am Heart Assoc.

[20] Aghemo A, Alekseeva OP, Angelico F (2022). Role of silymarin as antioxidant in clinical management of chronic liver diseases: a narrative review. Ann Med.

[21] Drawz P, Rahman M (2015). Chronic kidney disease. Ann Intern Med.

[22] Hopewell JC, Haynes R, Baigent C (2018). The role of lipoprotein (a) in chronic kidney disease. J Lipid Res.

[23] Enkhmaa B, Anuurad E, Berglund L (2016). Lipoprotein (a): impact by ethnicity and environmental and medical conditions. J Lipid Res.

[24] Lau YC, Proietti M, Guiducci E (2016). Atrial fibrillation and thromboembolism in patients with chronic kidney disease. J Am Coll Cardiol.

[25] Sacco RL, Kasner SE, Broderick JP, Caplan LR, Connors JJ, Culebras A (2013). An updated definition of stroke for the 21st century: a statement for healthcare professionals from the American Heart Association/American Stroke Association. Stroke.

[26] European Stroke O, Tendera M, Aboyans V, Bartelink ML, Baumgartner I, Clement D (2011). ESC guidelines on the diagnosis and treatment of peripheral artery diseases: document covering atherosclerotic disease of extracranial carotid and vertebral, mesenteric, renal, upper and lower extremity arteries: the task force on the diagnosis and treatment of peripheral artery diseases of the European Society of Cardiology (ESC). Eur Heart J.

[27] Halperin JL, Hankey GJ, Wojdyla DM, Piccini JP, Lokhnygina Y, Patel MR (2014). Efficacy and safety of rivaroxaban compared with warfarin among elderly patients with nonvalvular atrial fibrillation in the rivaroxaban once daily, oral, direct factor Xa inhibition compared with vitamin K antagonism for prevention of stroke and embolism trial in atrial fibrillation (ROCKET AF). Circulation.

[28] Johannesen CDL, Langsted A, Mortensen MB, Nordestgaard BG (2020). Association between low density lipoprotein and all cause and cause specific mortality in Denmark: prospective cohort study. BMJ.

[29] Tsimikas S, Gordts P, Nora C, Yeang C, Witztum JL (2020). Statin therapy increases lipoprotein(a) levels. Eur Heart J.

[30] Violi F, Pastori D, Pignatelli P (2014). Mechanisms and management of thrombo-embolism in atrial fibrillation. J Atr Fibrillation.

[31] O'Donoghue ML, Fazio S, Giugliano RP, Stroes ESG, Kanevsky E, Gouni-Berthold I (2019). Lipoprotein(a), PCSK9 inhibition, and cardiovascular risk. Circulation.

[32] Reyes-Soffer G, Ginsberg HN, Berglund L, Duell PB, Heffron SP, Kamstrup PR (2022). Lipoprotein(a): a genetically determined, causal, and prevalent risk factor for atherosclerotic cardiovascular disease: a scientific statement from the American Heart Association. Arterioscler Thromb Vasc Biol.

[33] Yan S, Li Q, Xia Z, Yan S, Wei Y, Hong K (2019). Risk factors of thromboembolism in nonvalvular atrial fibrillation patients with low CHA_2_DS_2_-VASc score. Medicine.

[34] Staerk L, Sherer JA, Ko D, Benjamin EJ, Helm RH (2017). Atrial fibrillation: epidemiology, pathophysiology, and clinical outcomes. Circ Res.

[35] Fless GM, Rolih CA, Scanu AM (1984). Heterogeneity of human plasma lipoprotein (a). Isolation and characterization of the lipoprotein subspecies and their apoproteins. J Biol Chem.

[36] Ferretti G, Bacchetti T, Johnston TP, Banach M, Pirro M, Sahebkar A (2018). Lipoprotein(a): a missing culprit in the management of athero-thrombosis?. J Cell Physiol.

[37] Tabas I, Williams KJ, Boren J (2007). Subendothelial lipoprotein retention as the initiating process in atherosclerosis: update and therapeutic implications. Circulation.

[38] Ezratty A, Simon DI, Loscalzo J (1993). Lipoprotein(a) binds to human platelets and attenuates plasminogen binding and activation. Biochemistry.

[39] Romagnuolo R, Marcovina SM, Boffa MB, Koschinsky ML (2014). Inhibition of plasminogen activation by apo(a): role of carboxyl-terminal lysines and identification of inhibitory domains in apo(a). J Lipid Res.

[40] Boffa MB, Koschinsky ML (2016). Lipoprotein (a): truly a direct prothrombotic factor in cardiovascular disease?. J Lipid Res.

[41] Boffa MB, Koschinsky ML (2019). Oxidized phospholipids as a unifying theory for lipoprotein(a) and cardiovascular disease. Nat Rev Cardiol.

[42] van der Valk FM, Bekkering S, Kroon J, Yeang C, Van den Bossche J, van Buul JD (2016). Oxidized phospholipids on lipoprotein(a) elicit arterial wall inflammation and an inflammatory monocyte response in humans. Circulation.

[43] Leibundgut G, Scipione C, Yin H, Schneider M, Boffa MB, Green S (2013). Determinants of binding of oxidized phospholipids on apolipoprotein (a) and lipoprotein (a). J Lipid Res.

[44] Edelstein C, Pfaffinger D, Hinman J, Miller E, Lipkind G, Tsimikas S (2003). Lysine-phosphatidylcholine adducts in kringle V impart unique immunological and potential pro-inflammatory properties to human apolipoprotein(a). J Biol Chem.

